# Frequent sputum production is associated with disturbed night’s rest and impaired sleep quality in patients with COPD

**DOI:** 10.1007/s11325-014-1111-9

**Published:** 2015-03-04

**Authors:** J. E. Hartman, J. Prinzen, R. C. van Lummel, N. H. T. ten Hacken

**Affiliations:** 1 Department of Pulmonary Diseases AA11, University of Groningen, University Medical Center Groningen, PO Box 30001, 9700 RB Groningen, The Netherlands; 2 GRIAC Research Institute, University of Groningen, University Medical Center Groningen, Groningen, The Netherlands; 3 McRoberts, Den Haag, The Netherlands

**Keywords:** Accelerometry, Chronic obstructive pulmonary disease, Night’s rest, Sputum production

## Abstract

**Purpose:**

In this study, we measured night’s rest parameters measured with an accelerometer and sleep quality in mild to very severe patients with COPD. Furthermore, our aim was to investigate the association between night’s rest parameters and clinical variables and the association between sleep quality and quality of life or health status.

**Methods:**

Mild to very severe COPD patients were recruited from general practitioners and outpatient clinics of general hospitals to participate in a cross-sectional study on physical activity in patients with COPD. A total of 103 patients (mean age 65 years, 67 % male) wore the accelerometer during night’s rest for at least four nights and were included in the analyses.

**Results:**

No significant associations were found between objectively measured body movements during night’s rest or subjective sleep quality and lung function, dyspnoea severity, body composition and physical activity during the day. Patients with frequent sputum production during the day had a higher number of sitting transitions during the night (5.3 vs 4.3 sitting transitions) and more frequently got out of bed compared to patients who hardly ever produced sputum during the day (1.0 vs 0.8 times per night). Furthermore, these patients also reported worse sleep quality (Pittsburgh sleep quality index (PSQI) score 4 vs 3).

**Conclusions:**

Our results indicate that objectively measured body movements during night’s rest like body postures and transitions are not related to sleep quality in patients with COPD. We did find an association between frequent sputum production and disturbances during night’s rest and sleep quality. Future studies should investigate whether the treatment of mucus hypersecretion leads to improved night’s rest.

## Introduction

Sleep problems are highly prevalent in patients with chronic obstructive pulmonary disease (COPD) [1]. Several studies have shown that sleep disturbances and impaired sleep quality are more prevalent in patients with COPD as compared with controls [2–4]. Potential causes for the high prevalence of sleep disturbances in this group of patients are higher age, pharmacotherapy, COPD-specific symptoms such as wheezing and cough, COPD-associated comorbidity (including sleep disorders), psychological distress due to COPD and hypoxemia. Importantly, low sleep quality has shown to be associated with low quality of life in patients with COPD [5, 6].

Sleep problems can be measured by subjective measures like questionnaires or objective measures like polysomnography, the golden standard for measuring sleep. Furthermore, accelerometers can be used to gain additional information on sleep/wake patterns and body movements during sleep (including body postures). An advantage of this objective measure is that it is cheap and can measure night’s rest at home for multiple consecutive nights. A recently published study investigated sleep with an accelerometer that was worn around the wrist for five to seven consecutive nights [3]. The authors concluded that objective information derived from accelerometers can be relevant for the evaluation and management of COPD patients [3]. This study only included 26 patients with COPD and no patients with mild COPD; thus, it would be useful to investigate night’s rest measured with an accelerometer in a larger and broader population of patients with COPD. Besides accelerometers worn around the wrist, there are devices that are worn around the waist. These devices are also able to measure body postures and to detect if patients go out of bed during the night, which could provide additional useful information on disturbances during night’s rest.

Only a few studies have investigated the association between night’s rest and important clinical outcomes of COPD. For example, one study found that impaired nocturnal sleep was related to the severity of dyspnoea [3]. Another interesting study showed that sleep quality measured with a questionnaire significantly improved after an 8-week pulmonary rehabilitation programme in COPD patients [7]. Which aspect of pulmonary rehabilitation was most effective in improving sleep quality in COPD patients was found to be uncertain; however, also in healthy individuals, higher exercise levels have been associated with better sleep quality [8]. Although sleep quality is an important outcome in COPD, little information is available about the impact of COPD-specific factors and the contribution to quality of life and how these factors interrelate. Therefore, it would be very interesting to investigate the association between physical activity during the day, dyspnoea severity and other clinical variables and night’s rest parameters more in-depth and in a large sample of COPD patients with mild to very severe COPD.

The aims of our study are to assess in mild to very severe patients with COPD (1) the level and variation of body movements during night’s rest measured with an accelerometer and sleep quality, (2) the association between night’s rest parameters and clinical variables and (3) the association between sleep quality and quality of life or health status.

## Methods

This study was part of a cross-sectional single-centre study on physical activity in people with COPD [9, 10]. Mild to very severe COPD patients were recruited from general practitioners and outpatient clinics of general hospitals in the northern part of The Netherlands. Participants were included in this study when they had a diagnosis of COPD according to the global initiative for obstructive lung disease (GOLD) criteria [11] (FEV_1_/FVC ratio post bronchodilator <0.7). Furthermore, comorbidity was allowed, but patients were excluded if they had a serious active disease that needed medical treatment (e.g. carcinoma) or were treated for a COPD exacerbation in the past 2 months. The study was approved by the ethics committee of the UMCG, and all patients gave written informed consent.

### Measurements

The participants performed all measurements during three study visits, including 7 days of 24 h accelerometer measurement.

#### Body movements during night’s rest measured with an accelerometer

Participants wore a triaxial accelerometer for 7 days, 24 h per day (DynaPort MoveMonitor, McRoberts). This accelerometer is a highly validated instrument for evaluating physical activity in patients with COPD [12, 13]. During the night, the device is able to estimate night’s rest start and night’s rest end. Any period of lying that lasts longer than 3 h, which is not interrupted for more than 15 min is detected as night’s rest. Furthermore, the device is able to detect if a person leaves the bed by analysing the interruptions of the night’s rest, this is called an ‘out-of-the-bed period’. The inclination of the trunk during lying is used to trace the subjects’ body postures which are categorized into ‘left side’, ‘right side’, ‘prone’ and ‘supine’. By calculating the size of the rotation vector, every movement is detected. Movement time is expressed as the percentage of the night’s rest that movement is detected. The average size of the rotation vector during nocturnal movement is used to calculate the intensity of all movement periods. The size of the rotation was measured by comparing the body posture before and after each movement. Each rotation of more than 10° is called a transition. A pilot study that validated the DynaPort accelerometer against another accelerometer and polysomnography concluded that the device is a valid measurement device for physical activity during sleep [14].

#### Sleep quality

Sleep quality was measured by the Pittsburgh sleep quality index (PSQI). The PSQI is a self-rated questionnaire that includes seven domains: subjective sleep quality, sleep latency, sleep duration, habitual sleep efficiency, sleep disturbances, use of sleeping medication and daytime dysfunction [15].

#### Clinical variables

##### Pulmonary function

Forced expiratory volume in 1 s (FEV_1_) and forced vital capacity (FVC) were measured using a spirometer (PFT, MasterScreen; Viasys) according to the European Respiratory Society/American Thoracic Society (ERS/ATS) guidelines [16]. Residual volume (RV) and total lung capacity (TLC) were measured by body plethysmography (PFT; Viasys) according to ERS/ATS guidelines [17].

*Physical activity during the day* was measured with the same triaxial accelerometer. Locomotion time was calculated by the sum of %walking and %shuffling during the day. Inactivity time was calculated by the sum of %lying and %sitting during the day.

*Body composition *Fat-free mass (FFM) was measured by bioelectrical impedance (Bodystat 1500) and calculated with COPD- and sex-specific equations [18].

*Dyspnoea severity* was registered by the modified Medical Research Council (mMRC) dyspnoea index [19].

*Sputum production* was measured by question 6 of the clinical COPD questionnaire (CCQ) [20].

#### Quality of life and health status

Quality of life was measured by a disease-specific questionnaire, the Saint George’s respiratory questionnaire (SGRQ) [21] and by a generic questionnaire, the RAND-36 [22]. Health status was measured by the CCQ [20].

#### Statistical analyses

In accordance with the literature [23], patients were included in the analyses if they had worn the accelerometer for at least four nights. A day was considered a valid measurement day if the device was worn for at least 94 % of the day [24]. Pearson or Spearman correlation coefficients were calculated to test univariate associations between night’s rest parameters and clinical variables. Differences between groups were tested with an independent sample *t* test, ANOVA, Mann-Whitney *U* test, Kruskall-Wallis or chi-square test. Nonparametric tests were performed in case of nonnormally distributed data. *p* values <0.05 were considered statistically significant. Statistical analyses were performed using IBM SPSS statistics (version 20).

## Results

### Subjects

**Fig. 1:**
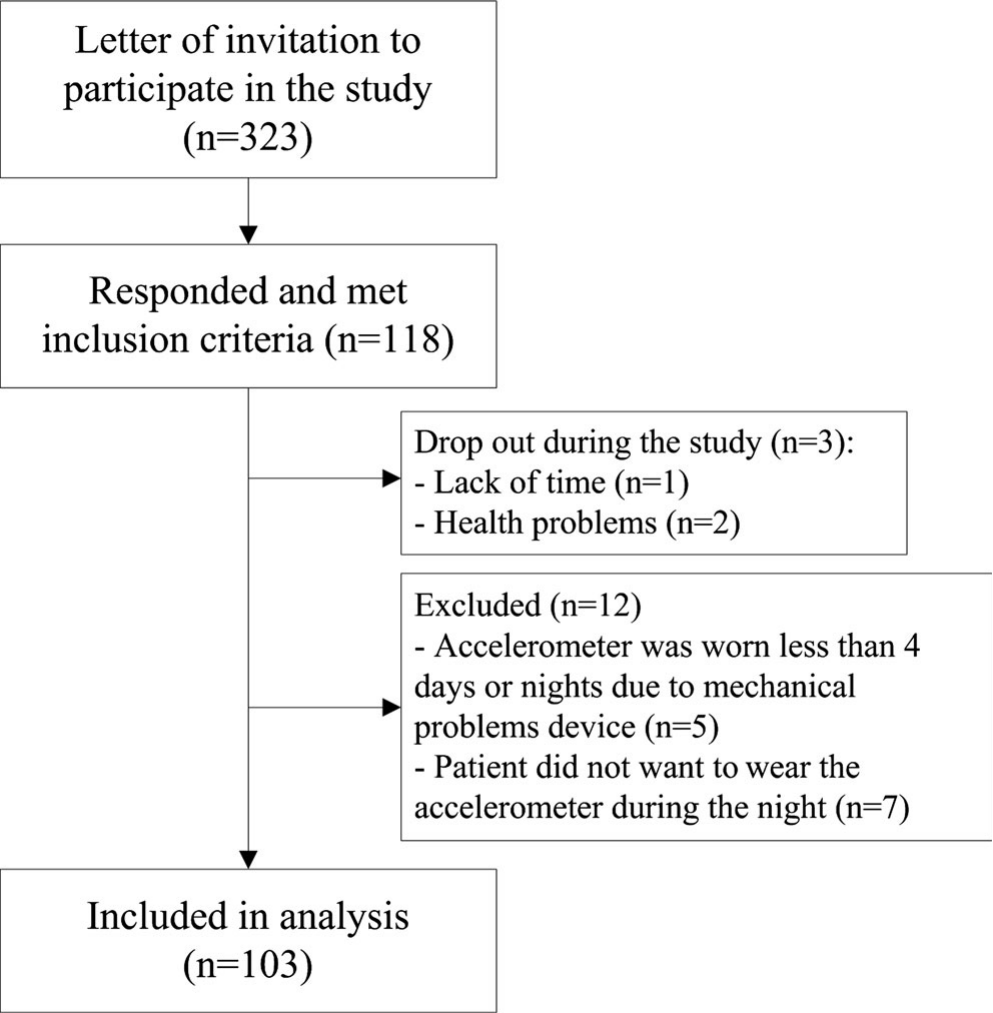
Flow of patients through the study

Of the 113 patients who wore the accelerometer during the study, 103 patients wore the accelerometer during night’s rest for at least four nights and were included in the analyses. Figure 1 shows the flow of participants through the study. Seven patients did not want to wear the accelerometer during the night, and 3 patients wore the accelerometer less than four nights due to technical problems with the device. Patient characteristics are shown in Table 1.

**Table 1:** Patient characteristics (*n* = 103)

Age, years		65.1 ± 8.7
Gender, male		69 (67.0 %)
Smoking status, current		29 (28.2 %)
LTOT user, yes		13 (12.6 %)
GOLD stage	I	28 (27.2 %)
	II	27 (26.2 %)
	III	29 (28.2 %)
	IV	19 (18.4 %)
FEV_1_, %predicted		52 (14–119)
BMI, kg/m^2^		25.1 ± 4.1
Education level, *n*	Low	65 (63.1 %)
	Middle	24 (23.3 %)
	High	14 (13.6 %)
Living situation, alone		26 (25.2 %)
Sputum production^a^, *n*	Hardly ever or never	68 (66 %)
	Frequently–always	35 (34 %)

Data are presented as mean ± standard deviation, number (percentage) or median (range)

*LTOT* long-term oxygen therapy, *GOLD* global initiative for obstructive lung disease, *FEV*_*1*_ forced expiratory volume in 1 s, *BMI* body mass index

^a^Sputum production was measured by question 6 of the COPD control questionnaire (CCQ). Hardly ever or never score = 0 or 1, frequently–always score = 2–6

### Level and variation of body movements during night’s rest and sleep quality

The results of the night’s rest parameters (both measured with the accelerometer and PSQI questionnaire) are shown in Table 2. The association between the start and end of the night’s rest measured by the accelerometer and reported by the patients in the PSQI questionnaire was high (rho = 0.64, *p* < 0.001 and rho = 0.73, *p* < 0.001, respectively). The night’s rest variables measured by the accelerometer were not associated with sleep quality measured by the PSQI, except for total number of transitions which was significantly but weakly associated with PSQI total score (rho = 0.21, *p* = 0.035). Furthermore, the night’s rest variables measured by the accelerometer were not significantly different between poor and good sleepers determined by the PSQI (poor sleepers’ PSQI total score ≥ 5).

**Table 2:** Night’s rest characteristics (*n* = 103)

Accelerometer		
Nights measured	4	1 (1.0 %)
	5	3 (2.9 %)
	6	12 (11.7 %)
	7	87 (84.5 %)
Start night’s rest, hour		23:44 (21:37–03:39)
End night’s rest, hour		8:17 (6:19–10:27)
Duration night’s rest, hour		8.22 ± 1.03
Times out of bed, *n*		0.9 (0–7.3)
Mean duration left side, %		32.9 (0–99.8)
Mean duration right side, %		45.2 (0–99.7)
Mean duration prone, %		0.0 (0–43.0)
Mean duration supine, %		13.8 (0–75.5)
Preferred body posture night’s rest	Left side	36 (35.0 %)
	Right side	56 (54.4 %)
	Prone	0
	Supine	11 (10.7 %)
Mean movement time, %		1.73 (0.4–8.91)
Mean movement intensity, g		0.062 ± 0.014
Mean transitions, *n* per hour		4.2 (1.3–11.7)
Sitting transitions during night, *n*		4.7 (2.0–16.30)
Pittsburg sleep quality index		
Start night’s rest, hour		23:30 (21.00–5.00)
End night’s rest, hour		8:00 (4:30–13:00)
Sleep onset latency, minutes		10 (0–180)
Night’s rest duration, hour		8.22 ± 1.13
Sleep duration, hour		7.5 (4–10)
PSQI, total score		4 (0–15)
Poor sleeper^a^, yes		28 (27.2 %)
Self-reported sleep quality	Very good	40 (38.8 %)
	Fairly good	48 (46.6 %)
	Fairly bad	15 (14.6)
	Very bad	0
Self-reported use of sleep medication	<1 per week	92 (89.3 %)
	≥1 per week	11 (10.7 %)
Trouble sleeping because of not breathing comfortably	<1 per week	90 (87.4 %)
	≥1 per week	13 (12.7 %)

Data are presented as mean ± standard deviation, number (percentage) or median (range)

*N* number, *g* average body acceleration, *PSQI* Pittsburgh sleep quality index

^a^PSQI ≥ 5 points

### Association between night’s rest parameters and clinical variables

The results of the association between night’s rest variables and clinical parameters are shown in Tables 3 and 4.

**Table 3:** Association between night’s rest parameters and clinical parameters (*n* = 103)

Accelerometer		Lung function	Physical activity during daytime	Demographics		Symptoms		
				Age	BMI	mMRC dyspnoea	Sputum production	
		FEV_1_ % pred	% locomotion				Hardly ever	Frequent
Start night’s rest		0.002	*−0.213*	0.074	0.128	0.064	*0:05*	*23:36*
End night’s rest		−0.003	*−0.224*	−0.049	−0.086	0.076	8:12	8:19
Duration night’s rest		0.009	0.012^a^	−0.073^a^	−0.089^a^	−0.031	8:13	8:29
Times out of bed		0.001	0.063	*0.258*	−0.039	−0.011	*0.8*	*1.0*
Sleep posture preference	Left side	50.0	10.06	65.56	25.68	2.0	23.3 %	43.3 %
	Right side	53.0	10.63	64.34	25.09	2.0	48.2 %	48.3 %
	Supine	68.0	10.87	67.09	23.69	2.0	14.0 %	8.3 %
Mean movement time		−0.025	0.004	−0.145	0.115	0.023	1.76	1.71
Mean movement intensity		0.167	*0.204* ^a^	−0.171^a^	−0.046^a^	−0.179	0.062	0.062
Sitting transitions during night		−0.065	−0.067	0.031	−0.032	0.100	*4.3*	*5.3*
PSQI questionnaire								
Sleep onset latency		−0.022	−0.073	−0.096	−0.184	−0.014	10.0	15.0
Sleep duration		−0.036	0.027	0.073	−0.022	0.081	8.0	8.5
PSQI total score		0.032	−0.018	−0.036	0.024	0.099	*3.0*	*4.0*
Sleep status	Poor sleeper	66.5	10.53	64.64	24.71	2.0	20.9 %	31.7 %
	Good sleeper	49.0	10.42	65.21	25.31	2.0	79.1 %	68.3 %
Self-reported sleep quality	Very good	51.0	9.98	66.85	25.45	2.0	48.8 %	31.7 %
	Fairly good	48.5	10.60	64.13	24.94	2.0	39.5 %	51.7 %
	Fairly bad	61.0	11.22	63.27	25.01	2.0	11.6 %	16.7 %
Use sleep medication	<1 per week	50.0	10.30	64.87	25.10	2.0	95.3 %	85.0 %
	≥1 per week	71.0	11.88	66.64	25.50	2.0	4.7 %	15.0 %

Data are presented as correlation coefficients or median (nonparametric) or mean (parametric). Correlation coefficients are Spearman’s rho. Differences between sleep posture preferences and self-reported sleep quality were tested with ANOVA (BMI, age and % locomotion) or Kruskall-Wallis (FEV_1_ % pred, mMRC dyspnoea). Differences between sleep status and use of sleep medication were tested with a *t* test (BMI, age and % locomotion) or Mann-Whitney *U* test (FEV_1_ % pred, mMRC dyspnoea). Differences between patients who hardly ever and frequently produce sputum between sleep posture preferences and self-reported sleep quality were tested with chi-square test. Differences between patients who hardly ever and frequently produce sputum were tested with Mann-Whitney *U* test, differences between duration night’s rest and mean movement intensity were tested with a *t* test. Statistically significant differences (*p* < 0.005) are indicated in italics

*PSQI* Pittsburgh sleep quality index

^a^Pearson correlation coefficient

**Table 4:** Association between night’s rest parameters and clinical parameters (*n* = 103)

Accelerometer		Lung function						Physical activity during daytime	Demographics
		FEV_1_/FVC	RV/TLC	GOLD stages					FFM index
				I	II	III	IV	% inactivity	
Start night’s rest		−0.018	−0.064	23:44	23:54	23:35	23:45	*0.212*	0.066
End night’s rest		0.023	0.027	8:28	8:01	8:17	8:07	*0.266*	−0.100
Duration night’s rest		0.078	0.048^a^	8:25	8:24	8:15	8:29	0.051^a^	−0.109^a^
Times out of bed		0.014	−0.072	0.9	0.7	1.2	0.7	0.048	0.020
Sleep posture preference	Left side	43.44	51.8	25.0 %	48.1 %	34.5 %	31.6 %	69.48	16.89
	Right side	47.32	49.9	60.7 %	44.4 %	55.2 %	57.9 %	67.76	16.83
	Supine	46.48	50.4	14.3 %	7.4 %	10.3 %	10.5 %	67.76	16.19
Mean movement time		−0.005	−0.002	1.68	1.73	1.98	1.68	−0.047	0.144
Mean movement intensity		0.172	*−0.208* ^a^	*0.064*	*0.067*	*0.057*	*0.06* ^b^	−0.124^a^	−0.071^a^
Sitting transitions during night		−0.034	0.049	4.75	4.0	5.1	4.7	0.154	−0.105
PSQI questionnaire									
Sleep onset latency		0.028	*0.204*	10.0	10.0	10.0	15.0	−0.005	−0.160
Sleep duration		0.045	0.124	8.0	8.5	8.25	8.75	0.048	−0.085
PSQI total score		0.115	0.143	4.0	4.0	3.0	4.0	−0.031	−0.044
Sleep status	Poor sleeper	47.46	53.1	39.3 %	25.9 %	24.1 %	15.8 %	66.74	16.92
	Good sleeper	42.14	49.7	60.7 %	74.1 %	75.9 %	84.2 %	68.99	16.42
Self-reported sleep quality	Very good	45.23	47.3	42.9 %	37.0 %	41.4 %	31.6 %	*70.53*	16.97
	Fairly good	42.05	52.3	42.9 %	40.7 %	41.4 %	68.4 %	*68.63*	16.88
	Fairly bad	47.42	54.0	14.3 %	22.2 %	17.2 %	0.0 %	*61.49* ^c^	16.00
Use sleep medication	<1 per week	44.60	50.3	85.7 %	88.9 %	86.2 %	100.0 %	68.94	16.83
	≥1 per week	54.29	53.7	14.3 %	11.1 %	13.8 %	0.0 %	62.96	16.42

Data are presented as correlation coefficients or median (nonparametric) or mean (parametric). Correlation coefficients are Spearman’s rho. Differences between sleep posture preferences and self-reported sleep quality were tested with ANOVA (RV/TLC, % inactivity and FFM index) or Kruskall-Wallis (FEV_1_/FVC). Differences between sleep status and use of sleep medication were tested with a *t* test (RV/TLC, % inactivity and FFM index) or Mann-Whitney *U* test (FEV_1_/FVC). Differences between GOLD stages between sleep posture preferences, self-reported sleep quality, sleep status and use of sleep medication were tested with chisquare test. Differences between GOLD stages were tested with Kruskall-Wallis, except for differences between duration night’s rest and mean movement intensity that were tested with ANOVA. Statistically significant differences (*p* < 0.05) are indicated in italics

*PSQI* Pittsburgh sleep quality index

^a^Pearson correlation coefficient

^b^GOLD stage II was significantly different compared to GOLD stage III

^c^Patients who reported very good sleep quality differed compared to patients who reported fairly bad sleep quality

*Lung function* There was no significant association between FEV_1_ %predicted or FEV_1_/FVC and body movement during night’s rest measured by the accelerometer or the PSQI. Only the mean movement intensity of movement during night was significantly different between patients with global initiative for obstructive lung disease (GOLD) stages II and III. No other significant differences were found between the different GOLD stages.

*Symptoms* We found no association between dyspnoea severity and the body movements during night’s rest measured by the accelerometer or PSQI. Patients who reported to never or hardly ever produce sputum (question 6 CCQ score 0 or 1) were compared with patients who reported to produce sputum several times to almost all the time (question 6 CCQ score 2–6). The patients who frequently reported to produce sputum had a significantly earlier start of the night’s rest, had a significantly higher number of times out of bed and a higher number of sitting transitions compared to the patients who reported to hardly produce sputum. Furthermore, the patients who reported to frequently produce sputum had a significant worse sleep quality score (PSQI total score).

*Body composition* There was no significant association between BMI or VVM index and body movement during night’s rest measured by the accelerometer or PSQI.

*Physical activity during the day* An earlier start of the night’s rest was significantly associated with higher percentage locomotion during the day and lower percentage inactivity during the day. An earlier end of the night’s rest was significantly associated with higher percentage locomotion during the day and lower percentage inactivity during the day. Furthermore, higher movement intensity during the night was significantly associated with higher percentage locomotion during the day.

### Association between sleep quality and quality of life or health status

The associations between sleep quality measured by the PSQI (total score) and quality of life domains measured by the different questionnaires are shown in Table 5. The PSQI total score was significantly associated with the mental health, vitality, bodily pain and health perception domains of the RAND-36; the symptom and mental state domains; and total score of the CCQ and none of the domains of the SGRQ. However, correlations were only weak or moderate (rho = 0.2–0.3).

**Table 5:** Univariate associations between PSQI total score and quality of life or health status questionnaires (*n* = 103)

	Correlation coefficient	*p* value
RAND-36		
Physical functioning	−0.067	0.503
Social functioning	−0.191	0.054
Role physical	−0.096	0.333
Role emotional	0.069	0.486
Mental health	*−0.268*	*0.006*
Vitality	*−0.278*	*0.004*
Bodily pain	*−0.317*	*0.001*
Health perception	*−0.288*	*0.003*
SGRQ		
Symptoms	0.113	0.258
Activity	0.100	0.315
Impacts	0.173	0.080
Total score	0.136	0.170
CCQ		
Symptoms	*0.227*	*0.021*
Functional state	0.170	0.087
Mental state	*0.210*	*0.033*
Total score	*0.218*	*0.027*

All Spearman’s rho

Statistically significant differences (*p* < 0.05) are indicated in italics

*SGRQ* St. George’s respiratory questionnaire, *CCQ* clinical COPD questionnaire

## Discussion

The results of our study show that patients with frequent sputum production during the day have a higher number of sitting transitions during the night and more frequently go out of bed compared to patients who hardly ever produce sputum during the day. Furthermore, these patients also reported worse sleep quality. Unexpectedly, objectively measured body movements during night’s rest were not associated with sleep quality. Furthermore, no significant associations were found between body movements during night’s rest measured by the accelerometer or sleep quality and lung function, dyspnoea severity, body composition and physical activity during the day.

The objectively measured body movements during night’s rest like body postures and transitions or going out of bed during the night were not significantly associated with self-reported sleep quality by COPD patients. This is in accordance with the findings of another study [3]. This indicates that these ‘disturbances’ during night’s rest do not automatically lead to poor sleep quality. Probably, sleep quality has a large individual subjective component.

Our results show that frequent production of sputum is associated with more disturbances during night’s rest and also with worse sleep quality. In our study, one third of the patients reported frequent sputum production during daytime. Therefore, ongoing mucus hypersecretion at night could potentially influence sleep in patients with COPD. It would be useful to investigate whether those patients indeed have increased sputum production at night. We expect that particularly, the chronic bronchitis patients have the increased sputum production and sleeping problems, but unfortunately, we have no data to discriminate between chronic bronchitis and emphysema patients. Our findings indicate that when a patient with COPD reports to have problems with sleep and to frequently produce sputum, this could be a treatment target. Potential treatments for mucus hypersecretion are smoking cessation [25] or drug therapy [26]. However, a randomized intervention study is necessary to investigate if the treatment of mucus hypersecretion improves sleep quality in patients with COPD.

We found that most night’s rest parameters were not significantly associated with clinical parameters. In accordance with other studies, night’s rest parameters were not significantly associated with lung function or GOLD stage [3, 5]; thus, a clear relationship between severity of airway obstruction and night’s rest is lacking. We did not find a clear significant association between physical activity during the day measured with the accelerometer and sleep parameters. This indicates that disturbances during night’s rest and sleep quality do not affect the physical activity level during the day. A qualitative study we performed in the same study population confirms this [10]. The results of this study showed that tiredness or poor sleep quality was not a frequently reported reason to be physically inactive [10]. In contrast to the study of Nunes et al. [3], who also measures sleep parameters with an accelerometer, we did not find a significant association between night’s rest parameters and dyspnoea severity measured by the mMRC scale. This study did not include patients with mild COPD and had a much higher percentage of poor sleepers according to the PSQI, which could explain the different results.

Our results showed that sleep quality was weakly to moderately significant associated with quality of life. Mainly, the more psychosocial domains of quality of life of the different questionnaires were significantly associated with sleep quality, while the more functional/physical domains were less often associated with sleep quality. These results indicate that self-reported sleep quality is more influenced by psychosocial factors than physical factors. Remarkably, the only health-related quality of life (HRQL) questionnaire that was not significantly associated with sleep quality was the COPD-specific HRQL questionnaire, the SGRQ. Two other studies did find an association between the SGRQ total score and sleep quality measured by the PSQI [5, 6]. However, both studies had much higher percentages of poor sleepers defined by the PSQI compared to our study.

A disadvantage of the study is that the accelerometer detects night’s rest and not sleep. Polysomnography does measure sleep and is able to detect sleep offset and efficiency and is the golden standard for sleep measurement. Nevertheless, the accelerometer is a cheap alternative that can easily measure important aspects of night’s rest at the patient’s home for multiple consecutive nights. Therefore, this instrument could be a useful alternative when polysomnography measurement is not indicated. In our study, only 7 out of 113 (6 %) patients did not want to wear the accelerometer during the night, and therefore, the feasibility appears to be good. Furthermore, the time patients themselves reported to go or get out of bed was highly associated with the start and end times of the night’s rest estimated based on the accelerometer data. Another potential drawback of our study is that we did not have a control group. It would be very useful to investigate if sleep problems and night’s rest parameters are different in patients with COPD compared to healthy controls. Another study did show that nocturnal sleep is impaired in patients with stable COPD compared to age-matched controls [3]. Unfortunately, this study had a small study population (*n* = 26), and it would be useful to include a larger control group. Remarkably, in our study, the percentage of patients that were identified as poor sleepers according to the PSQI questionnaire was much lower compared to other studies. In our study, 27 % of the patients were identified as poor sleepers while other studies reported 58–78 % [3, 5, 6, 27]. Therefore, our study population could be biassed as the percentage of poor sleepers is low. Alternatively, also a selection bias could be present in studies aimed to investigate sleep and therefore including a higher percentage of poor sleepers. Furthermore, the group sizes of the different GOLD stages are small in our study population and make it difficult to draw firm conclusions on the differences between disease severities. It would be useful to investigate the sleep quality of COPD patients in a large cohort study.

In conclusion, our results indicate that objectively measured body movements during night’s rest like sleep postures and transitions are not related to sleep quality in patients with COPD. We did find an association between frequent sputum production and disturbances during night’s rest and sleep quality. Future studies should investigate whether the treatment of mucus hypersecretion leads to improved night’s rest.

## References

[1] Agusti A, Hedner J, Marin JM, Barbe F, Cazzola M, Rennard S (2011). Night-time symptoms: a forgotten dimension of COPD. Eur Respir Rev.

[2] Bellia V, Catalano F, Scichilone N, Incalzi RA, Spatafora M, Vergani C, Rengo F (2003). Sleep disorders in the elderly with and without chronic airflow obstruction: the SARA study. Sleep.

[3] Nunes DM, de Bruin VM, Louzada FM, Peixoto CA, Cavalcante AG, Castro-Silva C, de Bruin PF (2013). Actigraphic assessment of sleep in chronic obstructive pulmonary disease. Sleep Breath.

[4] Valipour A, Lavie P, Lothaller H, Mikulic I, Burghuber OC (2011). Sleep profile and symptoms of sleep disorders in patients with stable mild to moderate chronic obstructive pulmonary disease. Sleep Med.

[5] Scharf SM, Maimon N, Simon-Tuval T, Bernhard-Scharf BJ, Reuveni H, Tarasiuk A (2010). Sleep quality predicts quality of life in chronic obstructive pulmonary disease. Int J Chron Obstruct Pulm Dis.

[6] Nunes DM, Mota RM, de Pontes Neto OL, Pereira ED, de Bruin VM, de Bruin PF (2009). Impaired sleep reduces quality of life in chronic obstructive pulmonary disease. Lung.

[7] Soler X, Diaz-Piedra C, Ries AL (2013). Pulmonary rehabilitation improves sleep quality in chronic lung disease. COPD.

[8] Montgomery P, Dennis J (2002) Physical exercise for sleep problems in adults aged 60+. *Cochrane Database Syst Rev* CD003404 10.1002/14651858.CD003404 PMC7017641 12519595

[9] Hartman JE, Boezen HM, de Greef MH, Ten Hacken NH (2013). Physical and psychosocial factors associated with physical activity in patients with chronic obstructive pulmonary disease. Arch Phys Med Rehabil.

[10] Hartman JE, ten Hacken NH, Boezen HM, de Greef MH (2013). Self-efficacy for physical activity and insight into its benefits are modifiable factors associated with physical activity in people with COPD: a mixed-methods study. J Physiother.

[11] Vestbo J, Hurd SS, Agusti AG, Jones PW, Vogelmeier C, Anzueto A, Barnes PJ, Fabbri LM, Martinez FJ, Nishimura M, Stockley RA, Sin DD, Rodriguez-Roisin R (2012). Global strategy for the diagnosis, management and prevention of chronic obstructive pulmonary disease, GOLD executive summary. Am J Respir Crit Care Med.

[12] Van Remoortel H, Raste Y, Louvaris Z, Giavedoni S, Burtin C, Langer D, Wilson F, Rabinovich R, Vogiatzis I, Hopkinson NS, Troosters T, on behalf of PROactive consortium (2012). Validity of six activity monitors in chronic obstructive pulmonary disease: a comparison with indirect calorimetry. PLoS One.

[13] Rabinovich RA, Louvaris Z, Raste Y, Langer D, Remoortel HV, Giavedoni S, Burtin C, Regueiro EM, Vogiatzis I, Hopkinson NS, Polkey MI, Wilson FJ, Macnee W, Westerterp KR, Troosters T, on behalf of the PROactive consortium (2013). Validity of physical activity monitors during daily life in patients with COPD. Eur Respir J.

[14] Bossenbroek L, Kosse N, Ten Hacken N, Gordijn M, Van der Hoeven J, De Greef M (2010). Validation of the DynaPort MiniMod during sleep: a pilot study. Percept Mot Skills.

[15] Buysse DJ, Reynolds CF, Monk TH, Berman SR, Kupfer DJ (1989). The Pittsburgh Sleep Quality Index: a new instrument for psychiatric practice and research. Psychiatry Res.

[16] Miller MR, Hankinson J, Brusasco V, Burgos F, Casaburi R, Coates A, Crapo R, Enright P, van der Grinten CP, Gustafsson P, Jensen R, Johnson DC, MacIntyre N, McKay R, Navajas D, Pedersen OF, Pellegrino R, Viegi G, Wanger J, ATS/ERS Task Force (2005). Standardisation of spirometry. Eur Respir J.

[17] Wanger J, Clausen JL, Coates A, Pedersen OF, Brusasco V, Burgos F, Casaburi R, Crapo R, Enright P, van der Grinten CP, Gustafsson P, Hankinson J, Jensen R, Johnson D, Macintyre N, McKay R, Miller MR, Navajas D, Pellegrino R, Viegi G (2005). Standardisation of the measurement of lung volumes. Eur Respir J.

[18] Steiner MC, Barton RL, Singh SJ, Morgan MD (2002). Bedside methods versus dual energy X-ray absorptiometry for body composition measurement in COPD. Eur Respir J.

[19] Bestall JC, Paul EA, Garrod R, Garnham R, Jones PW, Wedzicha JA (1999). Usefulness of the Medical Research Council (MRC) dyspnoea scale as a measure of disability in patients with chronic obstructive pulmonary disease. Thorax.

[20] van der Molen T, Willemse BW, Schokker S, ten Hacken NH, Postma DS, Juniper EF (2003). Development, validity and responsiveness of the Clinical COPD Questionnaire. Health Qual Life Outcomes.

[21] Jones PW, Quirk FH, Baveystock CM (1991). The St. George’s Respiratory Questionnaire. Respir Med.

[22] Aaronson NK, Muller M, Cohen PD, Essink-Bot ML, Fekkes M, Sanderman R, Sprangers MA, te Velde A, Verrips E (1998). Translation, validation, and norming of the Dutch language version of the SF-36 Health Survey in community and chronic disease populations. J Clin Epidemiol.

[23] Trost SG, McIver KL, Pate RR (2005). Conducting accelerometer-based activity assessments in field-based research. Med Sci Sports Exerc.

[24] Watz H, Waschki B, Meyer T, Magnussen H (2009). Physical activity in patients with COPD. Eur Respir J.

[25] Willemse BW, Postma DS, Timens W, ten Hacken NH (2004). The impact of smoking cessation on respiratory symptoms, lung function, airway hyperresponsiveness and inflammation. Eur Respir J.

[26] Kim V, Criner GJ (2013). Chronic bronchitis and chronic obstructive pulmonary disease. Am J Respir Crit Care Med.

[27] Shackell BS, Jones RC, Harding G, Pearse S, Campbell J (2007). ‘Am I going to see the next morning?’ A qualitative study of patients’ perspectives of sleep in COPD. Prim Care Respir J.

